# Free-Standing, Flexible Nanofeatured Polymeric Films Prepared by Spin-Coating and Anodic Polymerization as Electrodes for Supercapacitors

**DOI:** 10.3390/molecules26144345

**Published:** 2021-07-18

**Authors:** Guillem Ruano, Brenda G. Molina, Juan Torras, Carlos Alemán

**Affiliations:** Departament d’Enginyeria Química, Barcelona Research Center for Multiscale Science and Engineering, EEBE, Universitat Politècnica de Catalunya, C/Eduard Maristany 10-14, Edif. I2, 08019 Barcelona, Spain; guillem.ruano@upc.edu (G.R.); joan.torras@upc.edu (J.T.)

**Keywords:** conducting polymer, energy storage devices, nanoperforated films, polylactic acid, poly(3,4-ethylenedioxythiophene)

## Abstract

Flexible and self-standing multilayered films made of nanoperforated poly(lactic acid) (PLA) layers separated by anodically polymerized poly(3,4-ethylenedioxythiophene) (PEDOT) conducting layers have been prepared and used as electrodes for supercapacitors. The influence of the external layer has been evaluated by comparing the charge storage capacity of four- and five-layered films in which the external layer is made of PEDOT (PLA/PEDOT/PLA/PEDOT) and nanoperforated PLA (PLA/PEDOT/PLA/PEDOT/PLA), respectively. In spite of the amount of conducting polymer is the same for both four- and five-layered films, they exhibit significant differences. The electrochemical response in terms of electroactivity, areal specific capacitance, stability, and coulombic efficiency was greater for the four-layered electrodes than for the five-layered ones. Furthermore, the response in terms of leakage current and self-discharge was significantly better for the former electrodes than for the latter ones.

## 1. Introduction

Among energy storage devices, electrochemical supercapacitors (SCs) have gained *increasing attention* during recent years [1,2,3,4,5]. SCs exhibit higher power density and energy density than batteries and conventional capacitors, respectively. In SCs, electrodes are separated by an ion transport layer through which electrolyte ions shuttle to the electrode surfaces during the charging and discharging processes. Based on their different energy storage mechanisms, SCs are divided into several types: electrochemical double layer capacitors (EDLCs), pseudo-capacitors and hybrid capacitors [6,7]. For EDLCs, the electrical energy is stored by electrostatic accumulation of charges, while the energy storage in pseudo-capacitors is achieved through reversible and fast redox reactions. Hybrid capacitors are combinations of an EDLC or pseudo-capacitor electrode and a battery electrode in one SC. 

Independently of the energy storage mechanism, electrodes have a critical impact on the electrochemical performance of SCs and, therefore, their study deserves special attention. Within this context, a wide variety of electrode materials have been developed. For example, graphene [8,9,10], carbon nanotubes [11,12,13], and carbon nanofibers [14,15,16] are typical EDLC electrode materials, whereas metal oxides [17,18,19] and conducting polymers [20,21,22,23] (CPs) are pseudo-capacitor electrode materials. In general, pseudo-capacitor electrodes exhibit higher capacitance and energy density than EDLC electrodes. Instead, pseudo-capacitor electrodes show lower power density and rate capability than EDLC electrode materials. 

In recent years, flexible, lightweight, and environmentally friendly electrodes for SCs have attracted increasing attention since they meet the needs for portable (e.g., foldable phones) and wearable (e.g., smart textiles) electronics [24,25,26,27]. In these devices, which exhibit high specific energy and power densities and long life cycles, all components, including the electrodes, are flexible. In general, it is well-accepted that polymeric gel electrolytes fulfill the practical conditions required by the electrolyte layers of flexible SCs, especially in terms of electrochemical performance, excellent compressive/tensile properties, simple manufacturing properties, satisfactory tolerance over a wide temperature range [28,29,30,31]. However, the most suitable format for the electrodes in flexible SCs is still controversial and highly dependent on the final application of the device. Within this context, nanostructured conducting electrodes based on hydrogels [32,33,34], films [35,36,37], and fibers [38,39,40] have been reported for flexible SCs designed for different final applications.

In this work, we develop conducting and self-standing films of submicrometric thickness as flexible electrodes for SCs. These electrodes were obtained by alternating nanofeatured layers of an insulating polymer, which provided mechanical strength, and a CP that supplied electrochemical properties. Nanoperforations created in the insulating polymer layers were used to let the interpenetration of the CP layers, allowing the entire self-assembled film to be electrochemically active. Moreover, the two materials chosen for this device, polylactic acid (PLA) and poly(3,4-ethylenedioxythiophene) (PEDOT), are biocompatible, suggesting that flexible SCs prepared using such electrode are specially appropriated for biomedical applications. For example, the voltage and power consumption required by pressure sensors, radio transmitters, wearable sensor for biomolecules, pacemakers, and surface electromyography, are relatively low (i.e., typically <100 mV and <20 µW) and, therefore, could be supplied by flexible SCs. Furthermore, the combination of planarity, thinness, and flexibility provided by the electrodes reported in this work is particularly appropriated for applications requiring shape-adapted SCs. 

In comparison with other recent approaches used to prepare flexible films for energy storage applications [41], the strategy presented in this work has some relevant advantages. First, the nanofeatures in the PLA layer allows the incorporation of the CP layers by anodic polymerization (or electropolymerization) rather than by chemical methods, as, for example, oxidative polymerization. The former method favors the straight deposition of the CP film on the substrate, is very fast and efficient, and the layer thickness can be easily controlled by tuning the electrochemical parameters [42]. Furthermore, the combination of anodic polymerization and spin-coating provides a precise control on the thickness of the whole multilayered film, which is not easily achieved by other techniques [41]. Second, the relative distribution of the CP and PLA (i.e., external or internal layers) can be finely regulated and, even altered, through the applied approach, as is shown below.

Two different types of electrodes, which differ in the number of layers and, therefore, in the chemical nature of the external layer that can be of PLA (odd number of layers) or PEDOT (even number of layers), were prepared. More specifically, the system with an odd number of layers was obtained by self-assembling three PLA and two PEDOT layers alternatively (i.e., PLA/PEDOT/PLA/PEDOT/PLA), whereas the one with an even number of layer only contained two PLA layers (i.e., PLA/PEDOT/PLA/PEDOT). It should be remarked that the amount of electrochemically active CP is the same for the two electrodes, hereafter denoted 3PLA/2PEDOT and 2PLA/2PEDOT, respectively. Therefore, differences between them have been attributed to the effect of the external PLA layer that, although it was found to be beneficious for electromechanical (i.e., faradaic motors) [43] and tissue engineering applications [44], has a detrimental effect for energy storage applications.

## 2. Results

**Figure 1:**
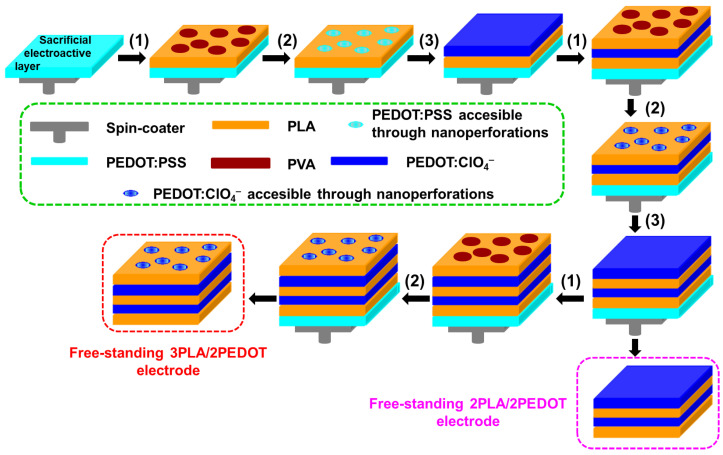
Descriptive scheme of the procedure used to prepare self-standing 3PLA/PEDOT and 2PLA/2PEDOT films.

The procedure used for the preparation of multilayered films formed by alternated layers of PLA and PEDOT was reported in previous work [43] and is schematically summarized in Figure 1. In brief, after spin-coating a sacrificial electroactive layer of PEDOT doped with polystyrene sulfonate (PEDOT:PSS) on a steel sheet (AISI 304) of 0.50 × 0.25 cm^2^, the rest of the process consisted in the sequential combination of three different steps. The first (steps “1” in Figure 1) was the spin-coating of a 90:10 *v*/*v* mixture of PLA and poly(vinyl alcohol) (PVA) solutions (both 10 mg/mL) in 1,1,1,3,3,3-hexafluoro-2-propanol (HFIP). After this, the second step involved the elimination of the nanospherical PVA domains by water-etching (steps “2” in Figure 1). It is worth noting that the diameter of such PVA nanofeatures, which were induced by the phase segregation between immiscible PLA and PVA during the spin-coating process, was adjusted to the thickness of the layer by selecting appropriated operational parameters (i.e., spinning speed, spinning time, and both concentration and solvent for the polymer solutions) [43,44]. Consequently, the electroactive material under the PLA layer became accessible through the nanoperforations obtained by removing PVA. Finally, the third step consisted in the electrochemical polymerization of PEDOT doped with ClO_4_^–^ anions (steps “3” in Figure 1). 

The formation of the PEDOT:ClO_4_^−^ layer was promoted by the electroactive material accessible through nanoperformations. For the fabrication of 3PLA/2PEDOT and 2PLA/2PEDOT films, steps “1” and “2” were repeated three and two times, respectively, while step “3” was repeated two times in both cases. Finally, the supported multilayered films were detached from the steel substrate by removing the PEDOT:PSS sacrificial layer, which was accomplished by submerging the supported membranes into milli-Q water for 12 h. The five- and four-layered films were completely detached from the steel with the help of tweezers, converting them into self-standing. Details about the operational parameters for the spin-coating and electropolymerization processes are described in the Electronic Supplementary Material.

**Figure 2:**
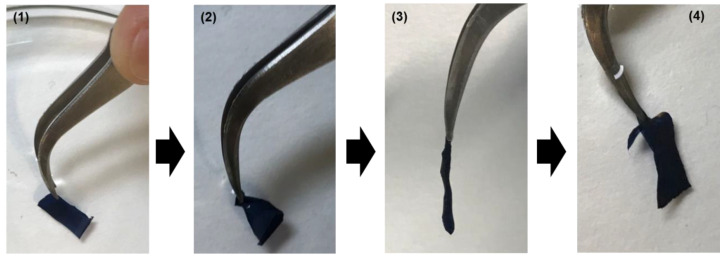
Digital camera photographs of a free-standing 2PLA/2PEDOT film: (**1**) floating on water; (**2**) clamped with tweezers while remains floating on water; (**3**) clamped out of water (in air); (**4**) introduced again on water.

Figure 2 shows photographs of self-supported 2PLA/2PEDOT films, which cannot be macroscopically distinguished from 3PLA/2PEDOT films (Figure S1). These self-standing films are very flexible and robust. This is evidenced in Figure 2, which show digital camera images of how the film floating in water folds on itself, experiencing a complete loss the shape when it exposed to air. However, the original shape is fully restored after the film is dropped back into the water. The thickness of the layers, as determined by perfilometry, was 45 nm (1st PLA), 259 nm (1st PEDOT), 94 nm (2nd PLA), 199 nm (2nd PEDOT), and 113 nm (3rd PLA).

**Figure 3:**
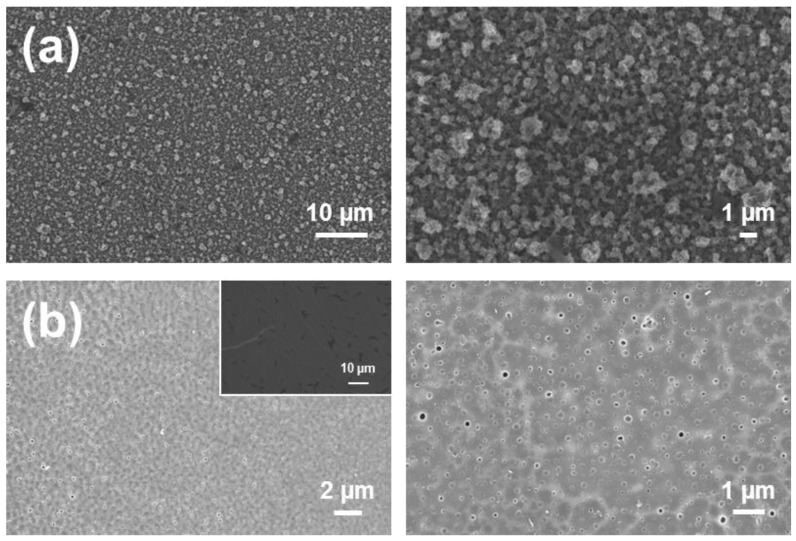
SEM micrographs of (**a**) 2PLA/2PEDOT and (**b**) 3PLA/2PEDOT.

The surface morphology of 2PLA/2PEDOT and 3PLA/2PLA films, which was studied by scanning electron microscopy (SEM), is displayed in Figure 3. 2PLA/2PEDOT surface is very similar to that previously described for single layered PEDOT films and can be described as a very homogeneous distribution of small aggregates, which are associated with the linear growing of polymer chains (Figure 3a) [20]. A completely different morphology was observed for 3PLA/2PEDOT (Figure 3b). Although low magnification SEM images suggest a compact, homogeneous, and flat surface, as is illustrated in the micrograph displayed in the inset, high magnification images evidence the presence of nanoperforations of 156 ± 23 nm in diameter. Moreover, the templating effect of the internal PEDOT layer on the external PLA layer is also reflected in the highest magnification micrograph (Figure 3b, right). Detailed description of the morphology and topography of 3PLA/2PLA films was provided in previous work [43,44] in which the effect of each layer was analyzed one-by-one.

**Figure 4:**
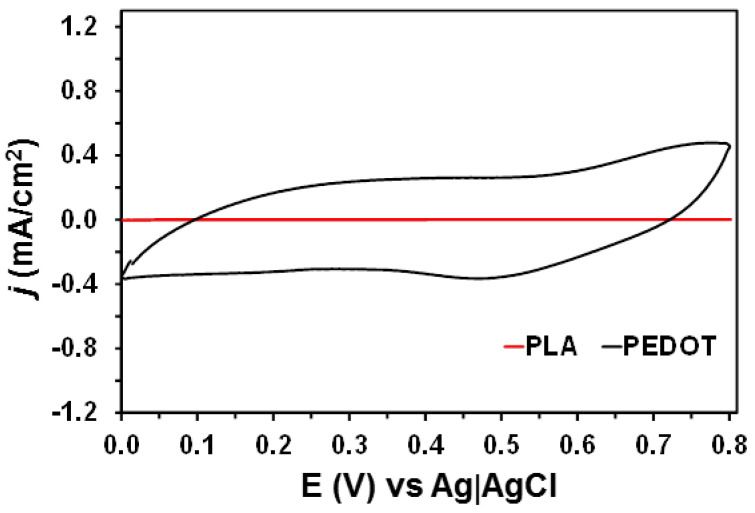
Cyclic voltammograms of pure PLA and pure PEDOT films, which were spin-coated and electropolymerized on steel electrodes, respectively, using a 0.1 M NaCl electrolytic solution. Initial and final potential: 0.0 V; reversal potential: 0.8 V. Scan rate: 100 mV/s.

Figure 4 compares the electrochemical behavior of pure PLA and pure PEDOT deposited on a steel electrode, which were spin-coated and electropolymerized, respectively, using the experimental conditions employed for the preparation of 2PLA/2PEDOT and 3PLA/2PEDOT. As it was expected, PLA was identified as an insulating and non-electroactive polymer, while PEDOT exhibited a very high electrochemical activity. According to these observations, the energy storage capacity discussed below for 2PLA/2PEDOT and 3PLA/2PEDOT must be exclusively attributed to PEDOT layers, while the contribution of PLA layers is related to both the flexibility and self-supporting behavior of multilayered films.

**Figure 5:**
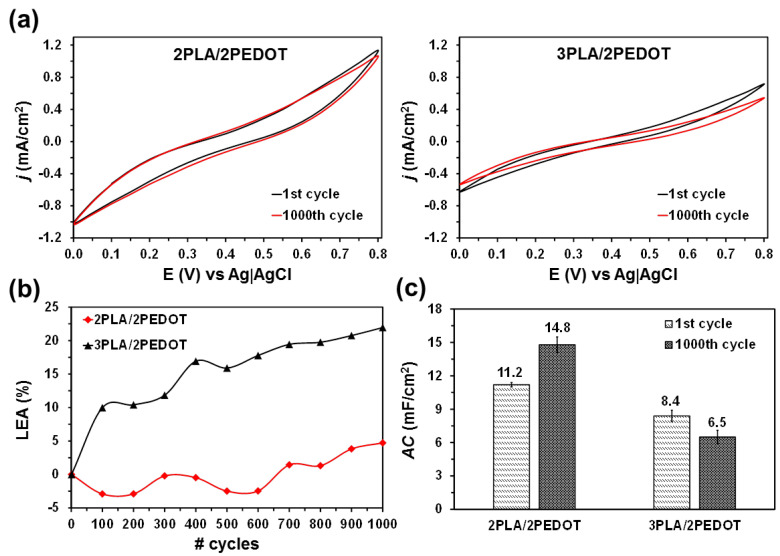
(**a**) Cyclic voltammograms of free standing (left) 2PLA/2PEDOT and (right) 3PLA/2PEDOT films using a 0.1 M NaCl electrolytic solution. Cyclic curves correspond to the 1st and 1000th cycles. Initial and final potential: 0.0 V; reversal potential: 0.8 V. Scan rate: 100 mV/s. (**b**) Evolution of the loss of electrochemical activity (LEA) with the number of redox cycles. (**c**) Areal specific capacitance (*AC*) determined for 2PLA/2PEDOT and 3PLA/2PEDOT electrodes after the 1st and 1000th voltammetric cycles.

Figure 5a compares the cyclic voltammetry (CV) control curves recorded of free-standing 2PLA/2PEDOT and 3PLA/2PEDOT electrodes in a 0.1 M NaCl solution. From a qualitative point of view, the non-quasi rectangular and symmetric shape of the two voltammograms is similar, indicating pseudocapacitive behavior and good reversibility, respectively [45,46,47]. The redox shoulders at 0.25 and 0.30 V are consistent with the high pseudocapacitive behavior. Another important characteristic of the voltammograms recorded for the two electrodes is the deviation from ideal horizontal voltammetric curves, which has been attributed to the presence of external and internal PLA layers in the films. From a quantitative point of view, the anodic and cathodic areas of the voltammograms are significantly larger for 2PLA/2PEDOT than for 3PLA/2PEDOT, indicating that the electrochemical activity (electroactivity) of the former is higher. Indeed, comparison of the total voltammetric charges, which are the sum of anodic and cathodic charge densities, indicates that the electroactivity is 61% higher for 2PLA/2PEDOT than for 3PLA/2PEDOT.

In order to evaluate the electrochemical stability of the electrodes, 1000 consecutive oxidation-reduction cycles were applied to both 2PLA/2PEDOT and 3PLA/2PEDOT using the same interval of potentials and scan rate. The voltammograms recorded after such 1000 CV cycles, which are included in Figure 5a, exhibit the same shape (i.e., non-quasi rectangular and symmetrical) that the control ones. However, the area varies, this feature being more evident for 3PLA/2PEDOT than for 2PLA/2PEDOT. This result suggests that the external layer of PLA is the most affected by the applied redox processes. 

Figure 5b represents the evolution of the loss of electrochemical activity (LEA; in %) against the number of CV cycles. The LEA was expressed as

(1) $$\mathrm{LEA}=\frac{Q_{i}-Q_{1}}{Q_{1}}\times 100$$

where *Q_i_* is the difference of voltammetric charge (in C) obtained for cycle *i* and *Q*_1_ is the voltammetric charge corresponding to the first cycle. For 3PLA/2EDOT the electrochemical activity decreases rapidly with increasing number of cycles. Thus, the LEA increases to 10% after only 100 cycles and, after that, the value progressively grows around 1% every 100 cycles. Conversely, the LEA of 2PLA/2PEDOT decreases up to −2.5% during the first 600 CV cycles, evidencing a self-stabilizing process. After that, the LEA increases by around 1% every 100 cycles. Comparison of the LEA profiles obtained for 3PLA/2EDOT and 2PLA/2EDOT confirms the previous hypothesis according to which the lower electrochemical stability of the former electrode is due to the damage induced by the potential scan in the last PLA layer. Figure S2 shows SEM micrographs proving that the apparition of surface defects (cracks) at the external layer of 3PLA/2PEDOT occurs after 100 cycles only.

The areal specific capacitance (*AC*; in mF/cm^2^) was determined using the following expression:

(2) $$AC=\frac{Q}{\Delta V\times A}$$

where *Q* is voltammetric charge determined by integrating the oxidative or the reductive parts of the cyclic voltammogram curve, Δ*V* is the potential window (in *V*), and *A* is the area of the electrode (in cm^2^). Results are compared in Figure 5c. The *AC* of 2PLA/2PEDOT electrode increases 15% after 1000 CV cycles (from 10.8 to 12.4 mF/cm^2^), which is consistent with the previously mentioned self-stabilizing effect, whereas that of 3PLA/2PLA decreases the same amount (from 6.4 to 5.6 mF/cm^2^). For the sake of completeness, specific capacitances, expressed as capacitance per gram of active PEDOT (F/g), are listed in Figure S3). As it can be seen, the specific capacitance of 2PLA/2PEDOT is as high as 452 ± 8 F/g increasing to 516 ± 24 F/g after 1000 CV cycles.

**Figure 6:**
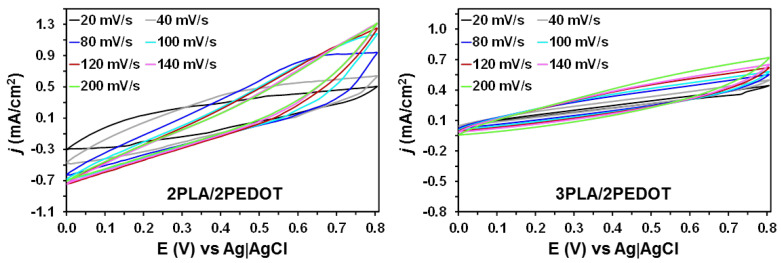
Cyclic voltammograms of (**left**) 2PLA/2PEDOT and (**right**) 3PLA/2PEDOT self-standing films using a 0.1 M NaCl electrolytic solution. Cyclic curves were determined using different scan rates, which range from 20 to 200 mV/s. Initial and final potential: 0.0 V; reversal potential: 0.8 V.

Energy storage ability of pseudocapacitive electrodes is typically related with the access and scape of the electrolyte into them. This diffusion-controlled process depends on the potential scan rate. Cyclic voltammograms of 2PLA/2PEDOT and 3PLA/2PEDOT films at different scan rates are shown in Figure 6. The deviation from the horizontal of the voltammograms increases with the scan rate. This loss of ideality indicates that the number of ions that successfully reaches the films decreases with increasing scan rate, since diffusion is limited. On the other hand, the unfavorable contribution of PLA layers to the electrical conductivity of the films explains the loss of the ideal rectangular shape, which is more evident for 3PLA/2PEDOT than for 2PLA/2PEDOT. All curves, with exception of that obtained for 3PLA/2PEDOT at the lowest scan rate, are symmetric, indicating good reversibility even at the higher rates.

**Figure 7:**
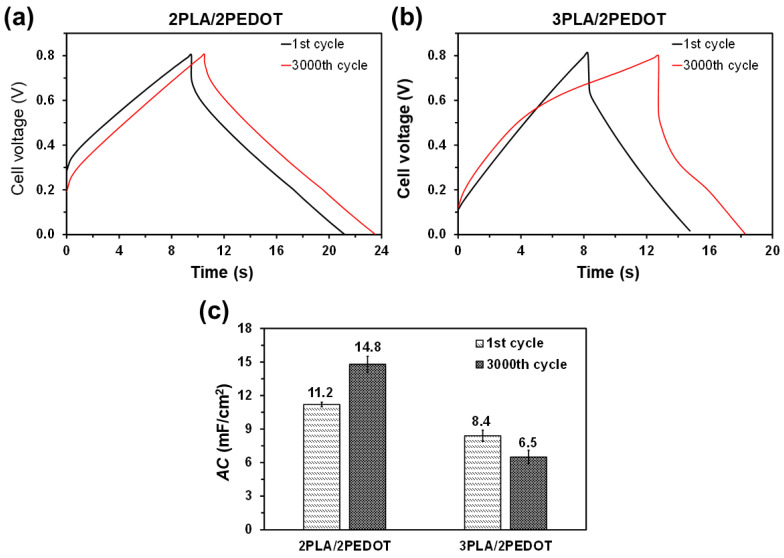
GCD cycles of free standing (**a**) 2PLA/2PEDOT and (**b**) 3PLA/2PEDOT films using a 0.1 M NaCl electrolytic solution. Curves correspond to the 1st and 3000th cycles. (**c**) *AC* determined for 2PLA/2PEDOT and 3PLA/2PEDOT electrodes after the 1st and 3000th GCD cycles.

Figure 7a,b shows the curve voltage versus time for galvanostatic charge-discharge (GCD) measurements performed at a 0.80 V window for a current density of 0.40 mA/cm^2^. Comparison of the triangle-like shaped GCD curves obtained for 2PLA/2PEDOT and 3PLA/2PEDOT indicates that charge and discharge times (tc and td, respectively) are longer for the former (tc = 9.5 s and td =9.5 s) than for the latter (tc = 8.2 s and td = 7.5 s). Indeed, a td shorter than tc, as observed for 3PLA/2PEDOT, indicates low coulombic efficiency (η). This parameter, defined as the ratio between td and tc, was of η = 1.0 and 0.9 for 2PLA/2PEDOT and 3PLA/2PEDOT, respectively.

After 3000 GCD cycles (Figure 7a,b), the value of tc increases to 10.5 and 13.5 s for 2PLA/2PEDOT and 3PLA/2PEDOT, respectively, while the td increases to 10.5 s for the former and decreases to 6.0 s for the latter. Accordingly, after 3000 GCD cycles, η remains at 1.0 for 2PLA/2PEDOT and decreases to 0.4 for 3PLA/2PEDOT. Consistently, the potential drop (Figure 7a,b) is twice for 3PLA/2PEDOT than for 2PLA/2PEDOT (i.e., 0.2 V vs. 0.1 V), indicating that the electrical conductivity is higher for the latter than for the former. Moreover, the potential drop of 2PLA/2PEDOT becomes almost inappreciable (i.e., <0.03 V) after 3000 GCD cycles. These results, which are fully consistent with CV observations, are corroborated by the AC (Figure 7c), which was 33% higher for 2PLA/2PEDOT than for 3PLA/2PEDOT after the first cycle, this difference increasing to 127% after 3000 GCD cycles. It should be noted that GCD cycles are much less aggressive than CV cycles. Thus, previous studies showed that potentiostatic redox cycles alter drastically the structure of polymers [48,49]. This feature explains that AC values derived from GCD measures are, in general, higher than those obtained using CV, as shown in Figure 5c and Figure 7c.

Overall, CV and GCD assays indicate that the connection between the two PEDOT layers increases with the number of cycles for 2PLA/2PEDOT, which has been attributed to the degradation of the internal PLA layer. Thus, the cleavage PLA chains at such internal layer probably results in charged species, giving place to additional parasitic electrochemical reactions and explaining the increment of the SC and the td, as well as the practical elimination of the potential drop, with the increasing number of cycles. On the contrary, the two external layers of PLA plug the internal layers of PEDOT (with the exception of nanoperforations) in 3PLA/2PEDOT, making electrochemical processes more difficult and, therefore, protecting the PLA chains of the internal layer from degradative oxidation and reduction processes.

**Figure 8:**
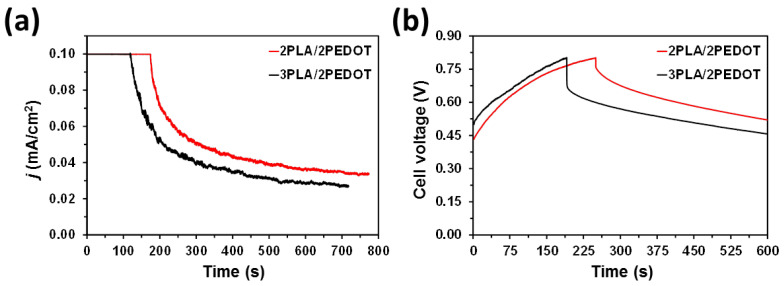
(**a**) Leakage current and (**b**) voltage drop of 2PLA/2PEDOT and 3PLA/2PEDOT after charging to 0.8 V at 0.1 mA.

Figure 8a compares the leakage current of 2PLA/2PEDOT and 3PLA/2PEDOT after charging to 0.8 V at 0.1 mA. As it can be seen, the discharge is not only faster for the former than for the latter but also the current stabilizes at a higher value for the former than for the latter. These feature indicate that 2PLA/2PEDOT electrodes provide better stability than 3PLA/2PEDOT ones. Figure 8b shows the voltage drop of charged systems. Such representative self-discharging curves indicate that the voltage of 2PLA/2PEDOT is systematically higher than that of 3PLA/2PEDOT. The rate of current leakage self-discharge is influenced by different factors, such as the chemistry and electrochemistry of the system, the purity of reagents and electrolyte and the temperature [50]. Considering that both types of electrodes were manufactured using identical chemical components (i.e., PLA, PEDOT) and the experimental conditions, the lower self-discharging tendency of 2PLA/2PEDOT reflects higher capacity to store energy electrochemically.

## 3. Methods and Materials

### 3.1. Materials

PEDOT: PSS 1.3 wt.% dispersion in water, 3,4-ethylenedioxythiophene (EDOT) monomer, PVA 87–89% hydrolyzed and lithium perchlorate (LiClO_4_) were purchased from Sigma-Aldrich (Saint Louis, MO, USA). LiClO_4_ was stored at 80 °C before its use. PLA 2002D pellets were supplied by Nupik International (Polinyà, Spain). Acetonitrile and HFIP were purchased from Panreac Quimica S.A.U. (Barcelona, Spain).

### 3.2. Preparation of the Films

Multilayered films were prepared by combining the spin-coating and the anodic polymerization techniques, following a recently reported procedure [43]. In brief, a steel sheet (AISI 304) of 0.50 × 0.25 cm^2^ was coated with a sacrificial layer of PEDOT:PSS by spin-coating deposition (1200 rpm for 60 s). Then, a PLA:PVA layer was generated onto the sacrificial layer by spin-coating (1200 rpm for 60 s) a 90:10 *v*/*v* mixture of PLA (10 mg/mL) and PVA (10 mg/mL) HFIP solutions. The nanoperforated PLA layer was obtained by removing the PVA domains via water etching. The resulting PEDOT:PSS/PLA bilayer was used as working electrode for the anodic polymerization of PEDOT doped with ClO_4_^−^ (PEDOT: ClO_4_^−^), as described below. Afterwards, the following nanoperforated PLA or PEDOT layers were obtained by iterating this procedure. Then, 5-and 4-layered films of composition PLA/PEDOT/PLA/PEDOT/PLA (3PLA/2PEDOT) and PLA/PEDOT/PLA/PEDOT (2PLA/2PEDOT), still supported onto the PEDOT:PSS-coated steel substrate, were achieved. These supported films were detached from the metallic substrate by selective elimination of the PEDOT:PSS sacrificial layer. This was achieved by submerging the supported films into milli-Q water for 12 h. Finally, films were completely detached from the steel substrate with the help of tweezers, and converted into self-standing multi-layered films. The spin-coating steps were performed using a WS-400BZ-6NPP/A1/AR1 (Laurell Technologies Corporation, North Wales, PA, USA) spin-coater.

PEDOT was prepared by anodic polymerization using an Autolab PGSTAT302N controlled by the NOVA software (Metrohm, Herisau, Switzerland). Polymerizations were carried out by chronoamperometry in a three-electrode cell filled with a 0.1 M LiClO4 acetonitrile solution containing 10 mM EDOT. In both cases, a constant potential of +1.40 V was applied and the polymerization charge was adjusted to 270 mC. The reference electrode was an Ag|AgCl electrode containing a KCl-saturated aqueous solution (E° = 0.222 V at 25 °C), while the counter electrode was a bare steel AISI 316L sheet.

### 3.3. Characterization

The morphology of the different samples was studied by scanning electron microscopy (SEM). Micrographs were acquired in a Focused Ion Beam Neon 40 (Zeiss, Oberkochen, Germany) equipped with an EDX spectroscopy system, operating at 5 kV, depending on the sensitivity to beam degradation of the studied systems.

Film thickness and roughness measurements were carried out using a Dektak 150 stylus profilometer (Veeco, Plainview, NY, USA). Conducted using the following settings: tip radius = 2.5 μm; stylus force = 3 mg; scan length = 1000 μm; speed = 33 μm s^−1^.

All electrochemical measurements were performed using a microcomputer-controlled potentiostat/galvanostat Autolab (Metrohm, Herisau, Switzerland) with PGSTAT101 equipment (Metrohm, Herisau, Switzerland) and the Nova software (Metrohm, Herisau, Switzerland). A conventional Ag|AgCl 3 M KCl electrode and a platinum electrode were used as reference electrode and counter electrode, respectively. 

The electrochemical response of 3PLA/2PLA and 2PEDOT/2PLA free standing films (0.50 × 0.25 cm^2^) as electrodes was studied in a three-electrode configuration by means of cyclic voltammetry (CV) and galvanostatic charge/discharge (GCD) measurements. The areal capacitance (*AC*) was calculated from CV cycles recorded in a three-electrode system (Equation (3)):

(3) AC=IA×(dVdt)

where *I* is the average discharge intensity, *A* is the surface area (0.125 cm^2^), and $\frac{dV}{dt}$ is the scan rate (100 mV/s). Furthermore, GCD measurements were also used to determine the cell capacitance, *AC*, by applying Equation (4).

(4) C=I×tdA×ΔV

where *I* is the applied current (5·10^−5^ A), *t_d_* is the discharge time, Δ*V* is the potential window (from 0.0 to 0.8V). The cycling stability of was tested by submitting the studied systems to *(i)* 3000 GCD cycles at a current density of 0.05 mA from 0.0 V to 0.8 V; and *(ii)* 1000 CV cycles at a scan rate of 100 mV/s from 0.0 V (initial and final potential) to 0.8 V (reversal potential). A 0.1 M NaCl solution was used as electrolyte in all cases.

The evaluation of the self-discharging (SD) and leakage current (LC) curves of SCs was carried out applying the following methodologies. In the first case, the SCs were charged to 0.8 V at 0.015 mA and kept at 1·10^−11^ mA for 10 min (i.e., self-discharging). After that time, the device was discharged to 0 V at −1 mA. In the second case, after charging the device to 0.8 V at 0.015 mA, it was kept at 0.8 V for 600 s while recording the current data through the SC (i.e., leakage current).

## 4. Conclusions

The performance as electrodes for SCs of multilayered films made of alternated nanometric layers of nanofeatured PLA and electrochemically polymerized PEDOT, has been evaluated. Both four- and five-layered films, which differ in the chemical nature of the external layer, which is PEDOT and nanoperforated PLA, respectively, have been prepared and characterized. In spite of the amount of conducting PEDOT is the same for both four- and five-layered films and electrochemical assays indicate that their performance as electrodes for energy storage devices is very different. Both CV and GCD assays show that the voltammetric charge and the stability of four-layered films is significantly higher than that of five-layered ones. Indeed, the AC is 69% and 33% higher for the former than for the latter as determined by CV and GCD, respectively. Moreover, four-layered films exhibit a self-stabilizing behavior with increasing number of cycles that is not detected in five-layered films. Indeed, the latter experiences a progressive loss of electroactivity with increasing number of cycles. Furthermore, four-layered electrodes exhibited the best performance in terms of current leakage and the self-discharging. In summary, results obtained for self-standing and flexible 2PLA/2PEDOT electrodes open new perspectives for their technological application in the biomedical and textile fields. 

## Data Availability

Data available upon request to authors.

## Supplementary Materials

The following are available online. Figure S1: Photograph of a free standing 3PLA/2PEDOT floating on water and clamped with tweezers; Figure S2: SEM micrographs showing the morphology (surface defects) at the external layer of 3PLA/2PEDOT after 100 cycles; Figure S3: Specific capacitance (in F/g) determined for 2PLA/2PEDOT and 3PLA/2PEDOT electrodes after the 1st and 1000th voltammetric cycles.

## Sample Availability

Samples of films are available from the authors upon request.
