# Supplementary material for: Free-Standing, Flexible Nanofeatured Polymeric Films Prepared by Spin-Coating and Anodic Polymerization as Electrodes for Supercapacitors
Source: Molecules. 2021 Jul 18;26(14):4345. doi: 10.3390/molecules26144345 (PMC8303661; doi:10.3390/molecules26144345)
Supplement: Supplementary file 1 [file molecules-26-04345-s001.zip › molecules-1287006-supplementary.pdf]

molecules

MDPI

Supplementary Information

Free-Standing, Flexible Nanofeatured Polymeric Films Prepared by Spin-Coating and Anodic Polymerization as Electrodes for Supercapacitors

Guillem Ruano, Brenda G. Molina *, Juan Torras and Carlos Alemán *

Departament d'Enginyeria Química and Barcelona Research Center for Multiscale Science and Engineering, EEBE, Universitat Politècnica de Catalunya, C/Eduard Maristany 10-14, Edif. I2, 08019 Barcelona, Spain; guil-lem.ruano@upc.edu (G.R.); joan.torras@upc.edu (J.T.)
* Correspondence: brenda.guadalupe.molina@upc.edu (B.G.M.); carlos.aleman@upc.edue (C.A.)

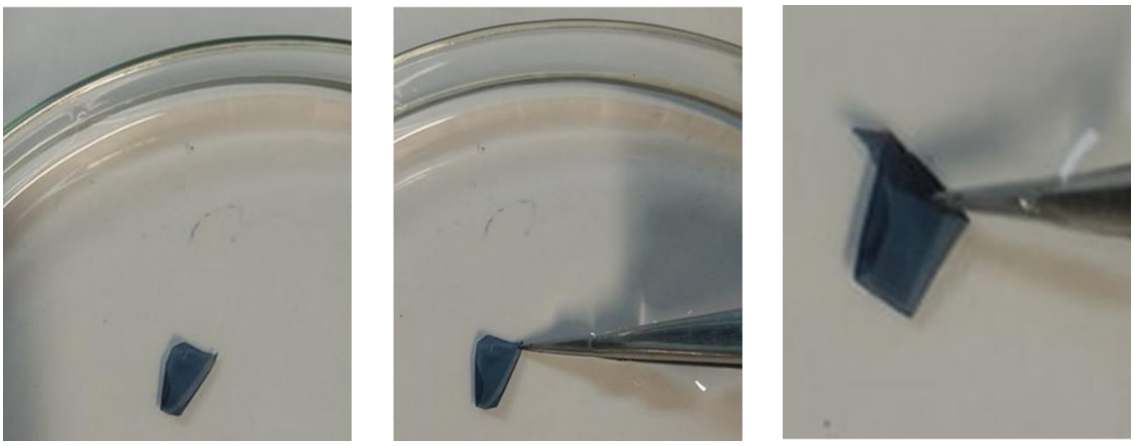

Figure S1. Photograph of a free standing 3PLA/2PEDOT floating on water and clamped with tweezers.

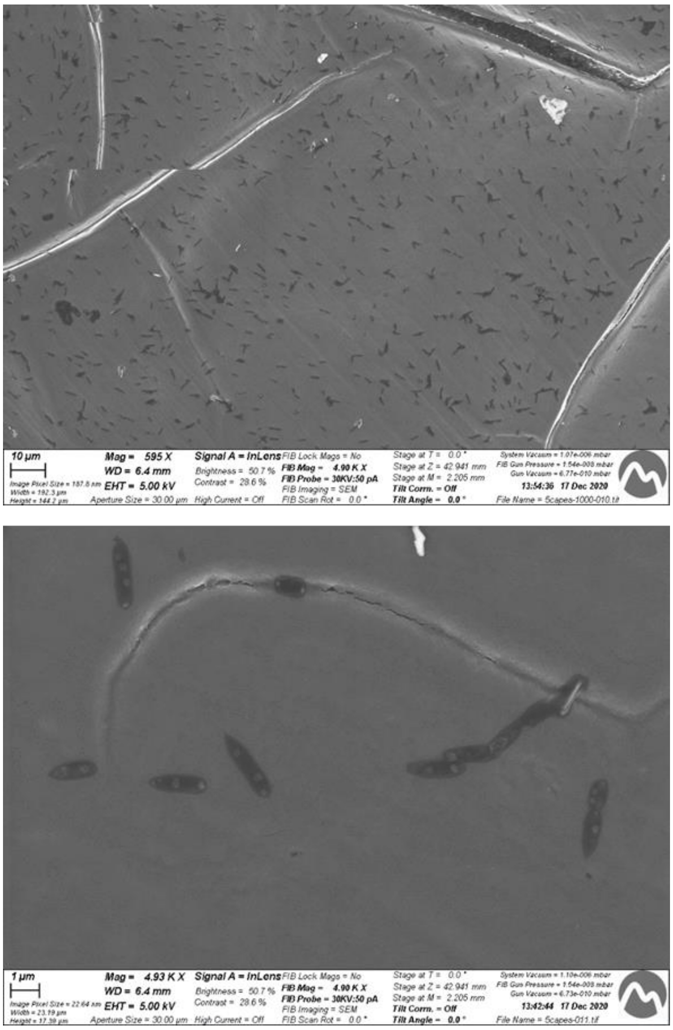

Figure S2. SEM micrographs showing the morphology (surface defects) at the external layer of 3PLA/2PEDOT after 100 cycles.

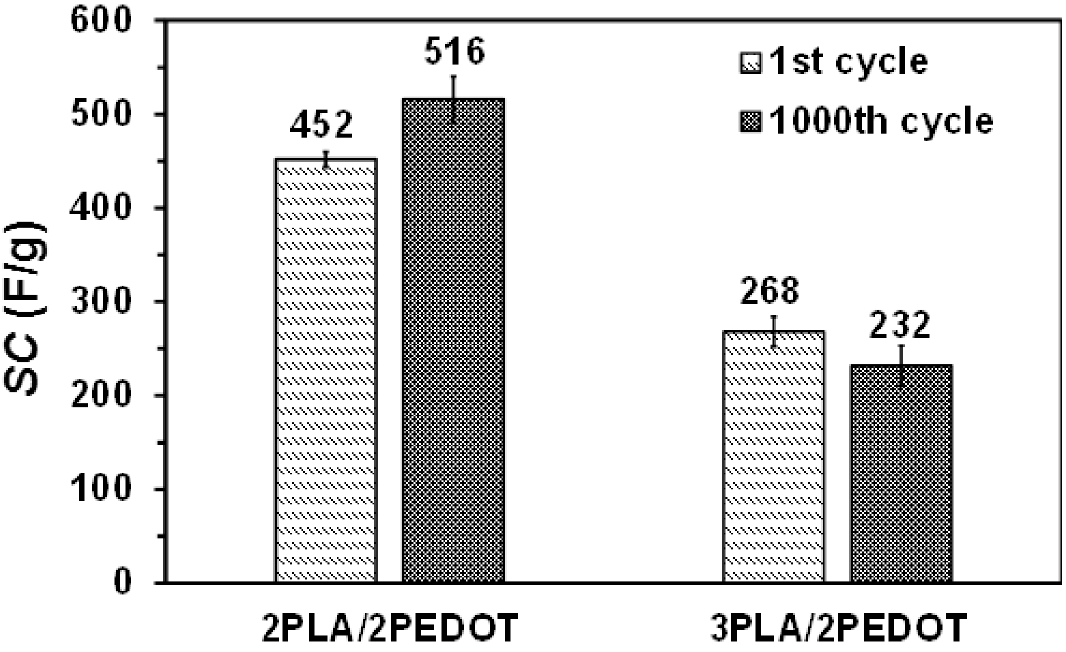

Figure S3. Specific capacitance (in F/g) determined for 2PLA/2PEDOT and 3PLA/2PEDOT electrodes after the 1st and 1000th voltammetric cycles.
